# Genes and Gene Networks Involved in Sodium Fluoride-Elicited Cell Death Accompanying Endoplasmic Reticulum Stress in Oral Epithelial Cells

**DOI:** 10.3390/ijms15058959

**Published:** 2014-05-20

**Authors:** Yoshiaki Tabuchi, Tatsuya Yunoki, Nobuhiko Hoshi, Nobuo Suzuki, Takashi Kondo

**Affiliations:** 1 Division of Molecular Genetics Research, Life Science Research Center, University of Toyama, 2630 Sugitani, Toyama 930-0194, Japan; 2 Department of Radiological Sciences, Graduate School of Medicine and Pharmaceutical Sciences, University of Toyama, 2630 Sugitani, Toyama 930-0194, Japan; E-Mails: yunokiki@med.u-toyama.ac.jp (T.Y.); kondot@med.u-toyama.ac.jp (T.K.); 3 Department of Animal Science, Graduate School of Agricultural Science, Kobe University, 1-1 Rokkodai, Kobe 657-8501, Japan; E-Mail: nobhoshi@kobe-u.ac.jp; 4 Noto Marine Laboratory, Institute of Nature and Environmental Technology, Kanazawa University, Housu-gun, Ishikawa 927-0553, Japan; E-Mail: nobuos@staff.kanazawa-u.ac.jp

**Keywords:** sodium fluoride, gene expression, gene network, cell death, endoplasmic reticulum stress, oral epithelial cell

## Abstract

Here, to understand the molecular mechanisms underlying cell death induced by sodium fluoride (NaF), we analyzed gene expression patterns in rat oral epithelial ROE2 cells exposed to NaF using global-scale microarrays and bioinformatics tools. A relatively high concentration of NaF (2 mM) induced cell death concomitant with decreases in mitochondrial membrane potential, chromatin condensation and caspase-3 activation. Using 980 probe sets, we identified 432 up-regulated and 548 down-regulated genes, that were differentially expressed by >2.5-fold in the cells treated with 2 mM of NaF and categorized them into 4 groups by *K*-means clustering. Ingenuity^®^ pathway analysis revealed several gene networks from gene clusters. The gene networks Up-I and Up-II included many up-regulated genes that were mainly associated with the biological function of induction or prevention of cell death, respectively, such as *Atf3*, *Ddit3* and *Fos* (for Up-I) and *Atf4* and *Hspa5* (for Up-II). Interestingly, knockdown of *Ddit3* and *Hspa5* significantly increased and decreased the number of viable cells, respectively. Moreover, several endoplasmic reticulum (ER) stress-related genes including, *Ddit3*, *Atf4* and *Hapa5*, were observed in these gene networks. These findings will provide further insight into the molecular mechanisms of NaF-induced cell death accompanying ER stress in oral epithelial cells.

## 1. Introduction

Fluoride is abundant in the environment and its addition to water and toothpaste has been instrumental in the decline of dental caries. A major source of fluoride is drinking water, including underground water contaminated by geological sources and fluoridated community drinking water. Although an appropriate range of fluoride is thought to be safe and effective for caries reduction, excessive fluoride intake results in toxic effects in either hard tissues of the teeth and skeleton or soft tissues of the kidneys, lungs and brain [1–4]. In addition, when toothpastes or mouse rinses are applied to the tooth surface, a relatively high concentration of fluoride comes into contact with and can be absorbed by the oral mucosa [5]. Previous reports have demonstrated that toxic effects of fluoride were observed in the oral mucosa *in vivo* [6,7] and oral cells *in vitro* [8–10].

Fluoride at a millimolar range affects diverse cellular functions such as enzyme activity, induction of DNA damage, signal transduction and cell-cycle changes [1,4,6,8,11], and induces forms of cell death, including apoptosis [1,6,8,11–13]. Sodium fluoride (NaF) is reported to be cytotoxic to oral mucosal fibroblasts due to its inhibition of protein synthesis and mitochondrial functions [8]. He and Chen [6] reported that fluoride could induce DNA damage and cell cycle changes and lead to apoptosis in oral mucosal cells and hepatocytes. In addition, NaF induces apoptosis through bcl-2 family protein-, caspase- and/or c-jun *N*-terminal kinase-mediated pathways [11,13].

The endoplasmic reticulum (ER) is a key organelle in the secretory pathway. ER stress occurs when unfolded or misfolded proteins are increased in the ER, triggering an unfolded protein response that is designed to restore protein homeostasis [14,15]. A variety of physiological and pharmacological insults lead to ER stress. The chaperone-binding protein, heat shock 70 kDa protein 5, glucose-regulated protein, and 78 kDa (Hspa5), a central regulator of ER homeostasis, are up-regulated under ER stress conditions [16]. The basic-region leucine zipper (bZIP) transcription factor DNA-damage-inducible transcript 3 (Ddit3) is induced in response to cellular stresses, especially ER stress, and is involved in the process of apoptosis associated with ER stress [17]. Experimental data have demonstrated that fluoride induces ER stress in several kinds of cells [9,10,18,19]. In ameloblast cells, fluoride elicits ER stress and expression of Hspa5 and inhibits cell growth and protein secretion [10,18]. Also, treatment with fluoride is reported to induce ER stress and apoptosis or cell damage and to increase the expressions of Hspa5 and Ddit3 [9,19]. As described above, many biological processes are affected by fluoride [1], whereas the overall responses to fluoride in cells are still unclear. Transcript profiling technologies including DNA microarray have made it possible to profile global gene expression patterns associated with different biological responses during chemical and physiological stresses. To date, several studies have utilized this technology for identifying changes in the gene expression of odontoblast culture cells induced by a fluoride at non-toxic doses [20], incisor pulp tissue during fluorosis [21] and the sperm of mice treated with fluoride [22]. Recently, pathway analysis technologies have enabled the mapping of gene expression data into relevant pathway maps on the basis of their functional annotation and known molecular interactions and we have used these in biological experiments [23,24]. The aim of the present study was to better understand the molecular mechanisms by which NaF induces cell death; thus, we determined the gene expression patterns of oral epithelial ROE2 cells exposed to this compound using a combination of global-scale microarrays and bioinformatics tools.

## 2. Results

### 2.1. The Effects of Sodium Fluoride (NaF) on Cell Number and Cell Viability in Oral Epithelial ROE2 Cells

**Figure 1.**
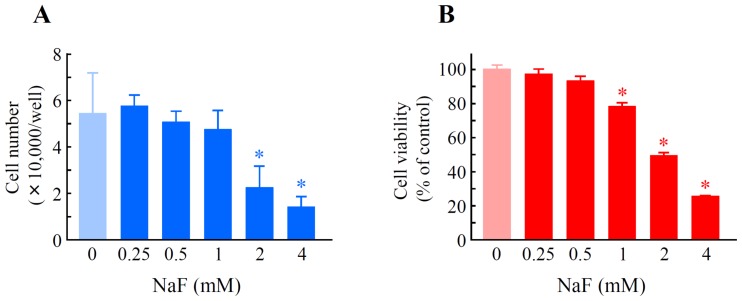
Effects of sodium fluoride (NaF) on cell number and cell viability in rat oral epithelial ROE2 cells. Cells were incubated with NaF at concentrations of 0 to 4 mM for 24 h. (**A**) Cell number was counted using a hematocytometer. The data represent the means ± standard deviations (*N* = 4); and (**B**) Cell viability was monitored using a WST-8 assay. Cells treated with 0 mM NaF served as a control (100%). The data represent the means ± standard deviations (*N* = 8). * *p* < 0.05 *vs.* NaF (0 mM) (Student’s *t*-test).

We monitored whether NaF affected cell number and cell viability in oral epithelial ROE2 cells [25]. NaF at concentrations of 2 and 4 mM resulted in a significant decrease in cell number by the trypan blue dye exclusion assay, and the inhibition percentages were 59.3% and 74.4%, respectively (Figure 1A). Exposure of the cells to NaF at concentrations of 1, 2 and 4 mM significantly and concentration-dependently decreased cell viability in the WST-8 assay, and the inhibition percentages were 22.3%, 50.5% and 74.7%, respectively (Figure 1B).

### 2.2. The Effects of NaF on Protein Content, Mitochondrial Membrane Potential (MMP) and Cell Death in ROE2 Cells

**Figure 2.**
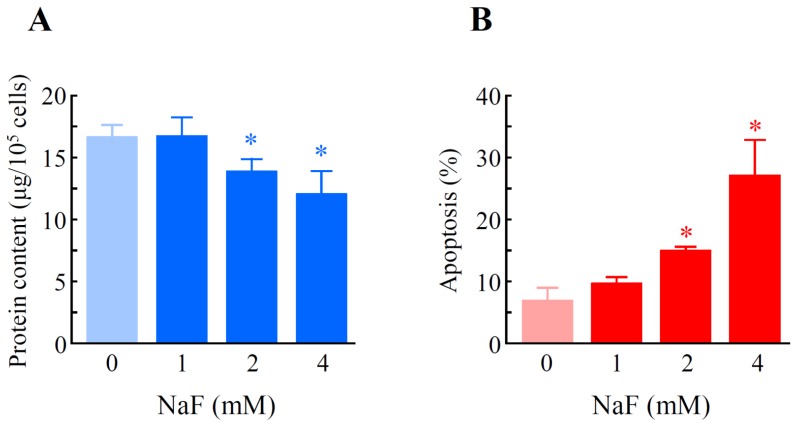
Effects of NaF on protein content and chromatin condensation in ROE2 cells. Cells were incubated with NaF at concentrations of 0 to 4 mM for 24 h. (**A**) The protein contents in cells were estimated by using a Pierce^®^ bicinchoninic acid (BCA) Protein Assay Kit (Pierce Biotechnology, Rockford, IL, USA); and (**B**) Chromatin condensation was measured using a Nuclear-ID Green Chromatin Condensation Kit (Enzo Life Sciences Inc., Farmingdale, NY, USA). The data represent the means ± standard deviations (*N* = 3).* *p* < 0.05 *vs.* NaF (0 mM) (Student’s *t*-test).

**Figure 3.**
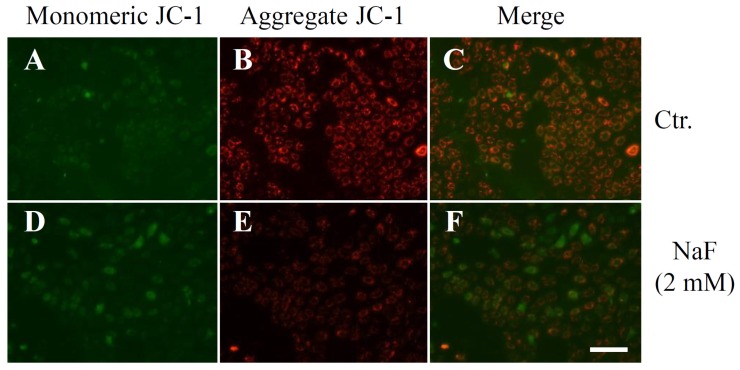
Effects of NaF on mitochondrial membrane potential (MMP) in ROE2 cells. Cells were cultured with or without 2 mM of NaF for 6 h. MMP was measured by JC-1, an indicator of mitochondrial function. (**A**,**D**) monomeric JC-1 green fluorescence; (**B**,**E**) aggregate JC-1 red fluorescence; and (**C**,**F**) merge images. (**A**–**C**) control cells (Ctr.); and (**D**–**F**) NaF-treated cells. Ctr., control. Scale Bar, 200 μm.

**Figure 4.**
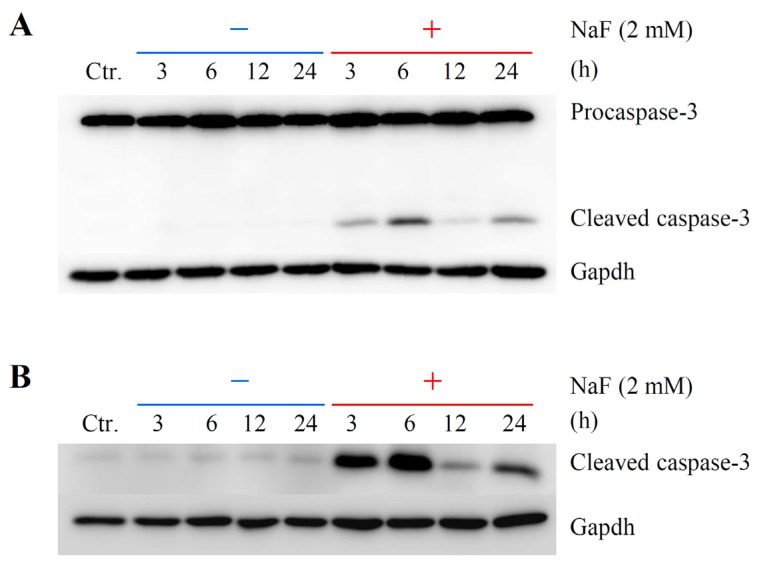
Effects of NaF on caspase-3 cleavage in ROE2 cells. Cells were cultured with or without 2 mM of NaF for 3 to 24 h. Western blot analysis was performed with anti-caspase-3 antibody, which reacted with procaspase-3 and cleaved caspase-3 (**A**); anti-cleaved caspase-3 antibody (**B**) and anti-Gapdh antibody. Gapdh served as a loading control. Ctr., control.

NaF (2 and 4 mM) significantly reduced the protein content in cells, and the inhibition percentages were 15.4% and 25.9%, respectively (Figure 2A). Mitochondrial membrane potential (MMP) was determined using JC-1, a cytofluorimetric dye. JC-1 can selectively enter into mitochondria and reversibly change color from green (the monomeric form of JC-1) to red (the aggregate form) as the membrane potential increases [26]. Although intact MMP (red fluorescence) was observed in the control group (Figure 3A–C), exposure of the cells to NaF (2 mM) induced a marked decrease of MMP (green fluorescence) (Figure 3D–F). When cell death was evaluated as the level of chromatin condensation, it was significantly increased to 15.0% and 27.1% in the cells treated with NaF at concentrations of 2 and 4 mM, respectively (Figure 2B). Caspase-3 is known to be a critical executioner of apoptosis, and is activated by the proteolytic processing of its inactive zymogen into activated p17 and p12 fragments [27]. A Western blot with anti-caspase-3 antibody clearly demonstrated that although the protein level of cleaved caspase-3 was below the detection limit in vehicle-treated cells, the level was markedly elevated in NaF (2 mM)-treated cells. The peak expression of the fragment was observed at 6 h after the treatment. On the other hand, the protein level of procaspase-3 was constant under both experimental conditions. Moreover, an increase in the expression level of cleaved caspase-3 was observed in compound-treated cells, which was demonstrated by Western blot with anti-cleaved caspase-3 antibody (Figure 4). These data indicated that NaF (2 mM) decreased either the protein content or MMP, and induced cell death. For further experiments, we selected a concentration of 2 mM NaF.

### 2.3. Global Gene Expression and Cluster Analyses

**Figure 5.**
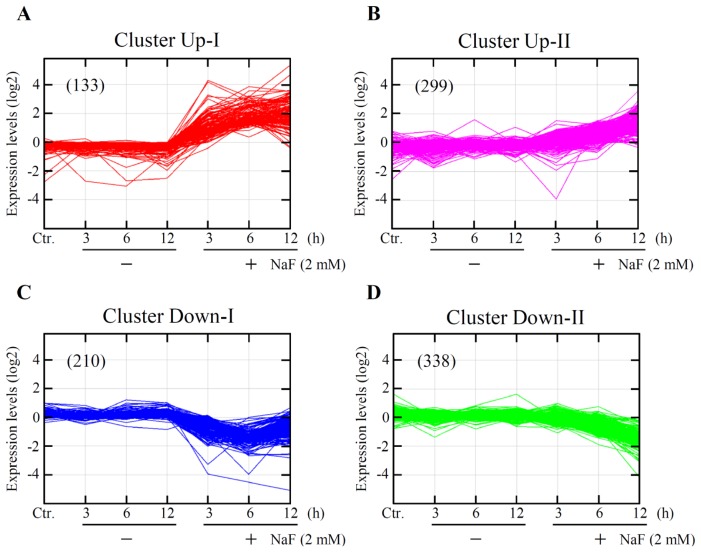
Cluster analysis of genes that were differentially expressed. ROE2 cells were cultured with or without 2 mM of NaF for 3 to 12 h. *K*-means clustering of the probe sets that were differentially expressed by a factor of 2.5 or more was carried out using the GeneSpring^®^ GX software (Agilent Technologies Inc., Santa Clara, CA, USA). Expression levels are shown. Non-treated cells served as a control (Ctr.). The figures in the parentheses indicate probe sets numbers. (**A**) cluster Up-I; (**B**) cluster Up-II; (**C**) cluster Down-I; and (**D**) cluster Down-II.

To identify genes that were differentially expressed and were involved in cell death induced by NaF in ROE2 cells, time course global-scale gene expression analysis was performed using a GeneChip^®^ system. Of the 31,099 probe sets analyzed, 11,379 probe sets were expressed in ROE2 cells treated with or without 2 mM of NaF for 3, 6 and 12 h treatments. The lists of probe sets obtained from cell samples have been deposited in the Gene Expression Omnibus, a public database, and are accessible through the series accession number (GSE53937). Expression analysis using GeneSpring^®^ software demonstrated that a total of 980 probe sets, 432 up-regulated and 548 down-regulated, were differentially regulated by a factor of 2.5 or more. In addition, *K*-means clustering, a non-hierarchical gene clustering algorithm, was conducted to generate the major patterns of gene expression during NaF-elicited cell death. As shown in Figure 5, the differentially expressed probe sets were classified in 4 clusters, designated as Up-I, Up-II, Down-I and Down-II. Clusters Up-I and Down-I contained 133 rapidly increased and 210 rapidly decreased probe sets, respectively. Clusters Up-II and Down-II contained 299 gradually increased and 338 gradually decreased probe sets, respectively. Information on the probe sets of these clusters is listed in Tables S1–S4 of the supplementary data.

### 2.4. Identification of Gene Functions and Gene Networks

**Figure 6.**
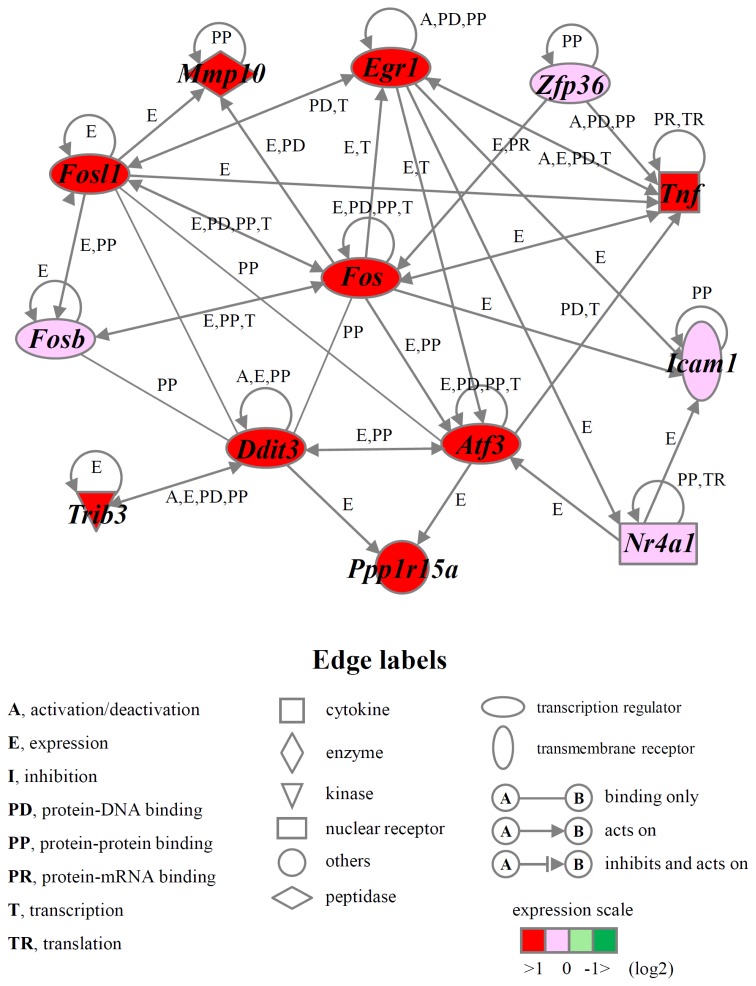
A gene network Up-I. Genes that were up-regulated in the cluster Up-I were analyzed by the Ingenuity^®^ Pathway analysis software. The network is displayed graphically as nodes (genes) and edges (the biological relationships between the nodes). Nodes and edges are displayed as various shapes and labels that present the functional class of genes and the nature of the relationship between the nodes, respectively.

**Figure 7.**
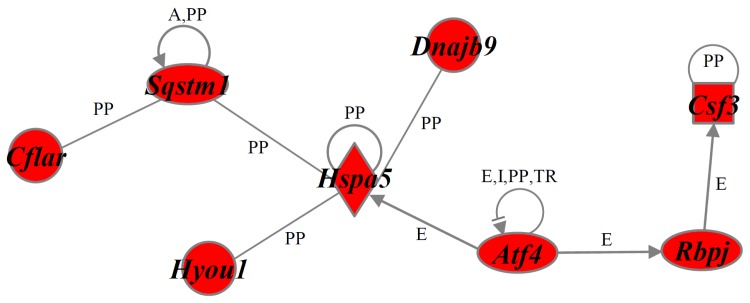
A gene network Up-II. Genes that were up-regulated in the cluster Up-II were analyzed by Ingenuity^®^ Pathway analysis software. For an explanation of the symbols and letters, see Figure 6.

To gain further insight into the molecular mechanism of NaF-induced cell death in ROE2 cells, we performed gene function and pathway analyses using the Ingenuity^®^ Pathways Knowledge Base (Ingenuity Systems Inc., Mountain View, CA, USA). The numbers of functionally annotated genes in the clusters Up-I (133 probe sets), Up-II (299), Down-I (210) and Down-II (338) were 58, 100, 103 and 158, respectively. Functional category analysis of genes of these clusters demonstrated many biological functions, including cell death or ER-stress. Table 1 shows the number of cell death-related genes whose functions were increased or decreased in Up-I and Up-II clusters. Moreover, ER stress-associated genes, such as *Ddit3* [17], *homocysteine-inducible*, *endoplasmic reticulum stress-inducible*, *ubiquitin-like domain member 1* (*Herpud1*) [28], *protein phosphatase 1*, *regulatory subunit 15A* (*Ppp1r15a*) [29] and *tumor necrosis factor* (*Tnf*) [30] (for Up-I) and *activating transcription factor 4* (*Atf4*) [31], *caspase 12* (*Casp12*) [32], *glucosidase β 2* (*Gba2*) [33], *heat shock protein 4-like* (*Hspa4l*) [34] and *Hspa5* [16] (for Up-II) were observed in these clusters. When cell death-associated genes belonging to clusters Up-I and Up-II were analyzed, the gene networks Up-I and Up-II were identified, respectively (Figures 6 and 7). The gene network Up-I contained many cell death-inducing genes, including *activating transcription factor 3* (*Atf3*) [35], *Ddit3* [17] and FBJ *osteosarcoma oncogene* (*Fos*) [36]. The gene network Up-II contained many cell death-preventing genes, including *Atf4* [37], *Hspa5* [38] and *sequestosome 1* (*Sqstm1*) [39].

### 2.5. Verification of Differentially Expressed Genes

**Figure 8.**
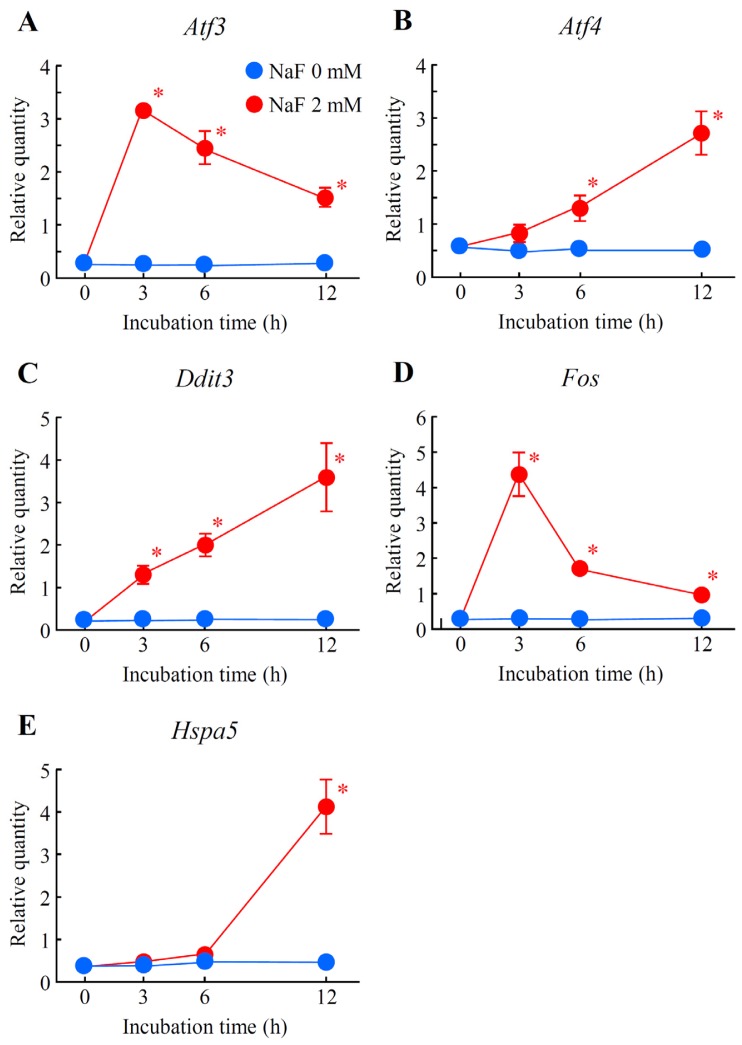
Verification of microarray results with real-time quantitative polymerase chain reaction (qPCR). Cells were incubated with or without NaF (2 mM) for 0 to 12 h. Real-time qPCR was performed. (**A**) *Atf3*; (**B**) *Atf4*; (**C**) *Ddit3*; (**D**) *Fos*; and (**E**) *Hspa5*. The data represent means ± standard deviations from 4 different experiments. Each expression level was normalized by Gapdh. Open circles, 0 mM NaF; closed circles, 2 mM NaF. * *p* < 0.05 *vs.* each NaF (0 mM) (Student’s *t*-test).

**Figure 9.**
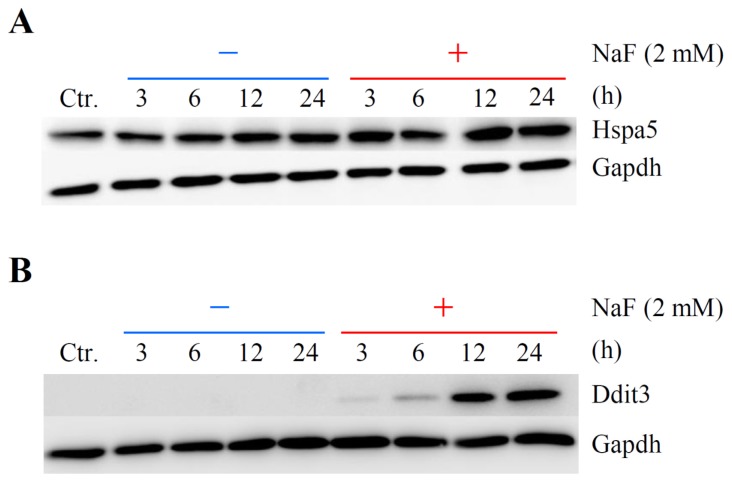
Effects of NaF on protein expressions for Hspa5 and Ddit3 in ROE2 cells. Cells were cultured with or without 2 mM of NaF for 3 to 24 h. Western blot analysis was performed with the specific primary antibodies for Hspa5 (**A**) and Ddit3 (**B**). Gapdh served as a loading control. Ctr., control (non-treated cells).

A real-time quantitative polymerase chain reaction (qPCR) assay was conducted to verify the microarray results. Five genes, *Atf3*, *Atf4*, *Ddit3*, *Fos* and *Hspa5*, were selected from the up-regulated genes that belonged to the gene networks Up-I and Up-II and were associated with cell death and/or ER stress. As demonstrated in Figure 8, the expression levels of 3 genes, *Atf3*, *Ddit3* and *Fos*, belonging to gene network Up-I were immediately and significantly up-regulated from 3 h after NaF (2 mM) exposure. Gradual and significant increases in the expressions of *Atf4* and *Hspa5* in gene network Up-II were detected. These data were almost comparable to the results of microarray analysis (Figure 8). Next, the expression levels of Hspa5 and Ddit3 proteins were monitored using Western blot analysis. The protein expression level of Hspa5 was constant in non-treated and vehicle-treated cells, but significantly elevated in the cells at 12 and 24 h after NaF (2 mM) treatment (Figure 9A). As shown in Figure 9B, although no expression of Ddit3 protein was detected in the control cells, the expression was significantly increased in the compound-treated cells in a time-dependent manner.

### 2.6. Effects of Knockdown of Hspa5 and Ddit3 on Cell Viability

**Figure 10.**
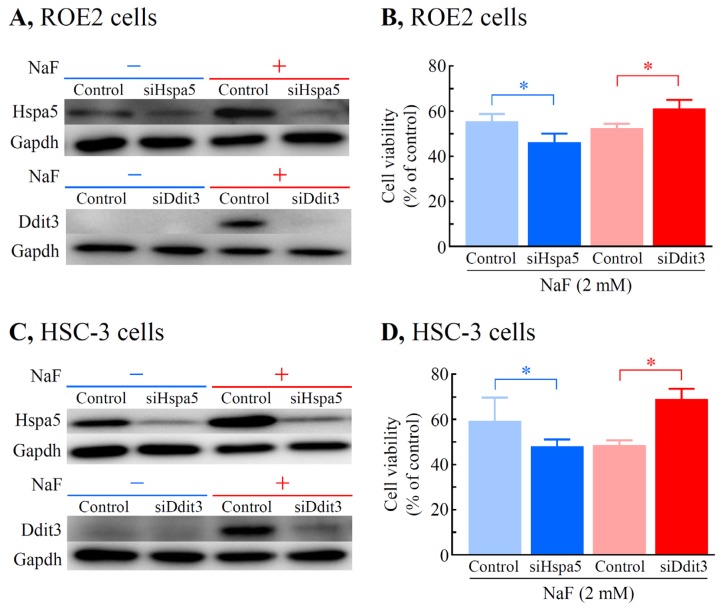
Effects of knockdown of Hspa5 and Ddit3 on the NaF-induced decrease in cell viability in rat oral epithelial ROE2 cells (**A**,**B**) and human malignant oral epithelial HSC-3 cells (**C**,**D**). Cells transfected with siRNA for *Hspa5*, *Ddit3* or *luciferase* were cultured with or without 2 mM of NaF for 24 h. Luciferase siRNA-treated cells served as a control. (**A**,**C**) Western blot analysis was performed with the specific primary antibodies for Hspa5 and Ddit3. Gapdh was used as a loading control; and (**B**,**D**) Cell viability was monitored using a WST-8 assay. Cell viability in the cells treated with 0 mM NaF was taken as 100%. The data represent the means ± standard deviations (*N* = 4–6). * *p* < 0.05 (Student’s *t*-test).

To knockdown genes such as *Hspa5* and *Ddit3*, RNA interference technology was used. As shown in Figure 10A, NaF (2 mM) induced significant increases in the protein expression levels of both Hspa5 and Ddit3 in rat oral epithelial ROE2 cells. On the other hand, treatment of the cells with the siRNAs for *Hspa5* and *Ddit3* effectively decreased the NaF-induced increases in the Hspa5 and Ddit3 protein expressions, respectively. In addition, silencing of either Hspa5 or Ddit3 significantly reduced or elevated the cell viability, respectively, to 46.0% (*vs.* 55.0% in the control) or 60.4% (*vs.* 51.9% in the control) (Figure 10B). Next, the roles of these proteins in the human malignant oral epithelial cell line HSC-3 were evaluated. NaF at a concentration of 2 mM elicited a marked elevation in the protein expression levels of Hspa5 and Ddit3 compared with control cells. When the siRNAs for *Hspa5* and *Ddit3* were transfected into HSC-3 cells, effective silencing of these gene products was observed (Figure 10C). In HSC-3 cells, knockdown of Hspa5 significantly reduced the cell viability to 46.6% *vs.* 59.0% in control cells. In contrast, *Ddit3*-knockdown significantly increased the cell viability to 68.3% *vs.* 47.9% in the control group (Figure 10D). These data demonstrated that *Hspa5* and *Ddit3* exerted cytoprotective and cytodamaging effects, respectively, in both cell lines exposed to NaF.

## 3. Discussion

The oral application of toothpastes and mouth rinses results in relatively high fluoride levels in the oral cavity. In fact, fluoride toxicity has been reported in both *in vivo* [6,7] and *in vitro* oral mucosal models [8–10]. It is also well known that fluoride at a millimolar range elicits complex cellular responses such as enzyme activity, signal transduction, ER stress and cell death in a wide variety of cell types [1]. Because of the complex cellular responses induced by fluoride, we thought that a combination of array-based transcript profiling with bioinformatics analysis technologies was the most useful approach for elucidating the molecular mechanisms of fluoride. This approach, as used in the present study, was the first to demonstrate the genes and gene networks involved in the cell death accompanying ER stress induced by NaF in oral epithelial cells.

In the present study, cell death measured as chromatin condensation was induced by relatively high concentrations of NaF (2 and 4 mM) in rat oral epithelial ROE2 cells. The induction of apoptosis by this compound was confirmed by the activation of caspase-3. In addition, NaF inhibited the protein synthesis and decreased MMP in ROE2 cells as previously reported in oral mucosal fibroblasts [8]. These results were comparable to those in previous studies [1,6,8,11–13]. Global-scale microarray and computational gene expression analyses clearly demonstrated that many probe sets that were differentially expressed by a factor of 2.5-fold or greater could be categorized into 4 gene clusters with distinct expression patterns. Interestingly, we further identified a number of genes and several gene networks associated with cell death from each gene cluster by using the Ingenuity^®^ Pathways Knowledge Base. Moreover, these gene clusters included many ER stress-related genes such as *Ddit3* [17] and *Hspa5* [16]. In our cell system exposed to NaF, the remarkable elevations of the expressions of *Ddit3* and *Hspa5* were verified by real-time qPCR and Western blotting, suggesting that NaF induces ER stress as described in previous manuscripts [9,10,18,19]. In this study, ER stress-related genes were observed in up-regulated gene clusters rather than down-regulated gene clusters. Therefore, we focused on either the up-regulated genes or gene clusters Up-I and Up-II. It was of particular interest that, among the 133 rapidly increased probe sets (gene cluster Up-I), the gene network Up-I consisting of 13 genes was principally associated with the biological function of increased cell death (Figure 6). This gene network included several bZIP transcription factors, such as *Atf3* [35], *Ddit3* [17], *Fos* [36], FBJ osteosarcoma oncogene B (*Fosb*) [40], and *fos-like antigen 1* (*Fosl1*) [41]. Of these, Ddit3 has been reported to be a key molecule in the induction of apoptosis during ER stress [17]. As expected, gene silencing experiments using siRNA for *Ddit3* demonstrated that Ddit3 had a role in the induction of cell death induced by NaF in the oral epithelial cell lines ROE2 and HSC-3. It has been suggested that homo- and hetero-dimeric protein complexes of the bZIP protein act as activators and repressors of transcription [42]. In the gene network Up-I, relationships have been identified between *Ddit3* and each of *Atf3*, *Fos*, *Fosb* and *Fosl1* [42,43]. In addition, previous studies demonstrated that *Ppp1r15a* and *tribbles pseudokinase 3* (*Trib3*) are targets of *Ddit3* and are involved in *Ddit3*-dependent apoptosis accompanying ER stress [29,44,45]. In addition, increased apoptotic effects of intercellular adhesion molecule 1 (Icam1) [46], matrix metallopeptidase 10 (Mmp10) [47], nuclear receptor subfamily 4, group A, member 1 (Nr4a1) [48], Tnf [49], and zinc finger protein 36 (Zfp36) [50] have been documented. In the present work, we further uncovered the gene network Up-II (8 genes), which was associated with the cell death-preventing function, from the gene cluster Up-II that contained 299 gradually increased probe sets (Figure 7). In NaF-treated ROE2 cells, increases in the expression levels of *Atf4* and *Hspa5* were confirmed by real-time qPCR. Under ER stress conditions, ATF4 activates the transcription of *Hspa5* [14,15] and this gene product is reported to inhibit apoptosis [37,38]. In the present study, cytoprotective effects of Hspa5 were observed in our cell systems treated with NaF. Under both gene clusters Up-I (13 genes) and Up-II (8 genes), 6 genes, *Atf4* [51], *Ddit3* [9], *Fos* [52], *Hspa5* [9], *Icam1* [46] and *Tnf* [53], out of 21 genes were shown to be increased under apoptotic conditions induced by NaF. However, a large portion of the differentially regulated genes represented newly identified genes whose relationships to the effects of NaF have not yet been reported. The differentially expressed genes identified here, and/or the gene networks they act within, are likely to be involved in cell death accompanying ER stress induced by NaF in oral epithelial cells. However, the relationship between gene expression and cell death and/or ER stress is unknown. Further investigations will be required to clarify this issue.

Several studies have investigated the effects of fluoride on gene expression using microarray technologies. Although Wurtz *et al.* [20] demonstrated 8 differentially expressed genes in odontoblast culture cells induced by a fluoride at a non-toxic dose (1 mM), these were not detected in genes that were identified in our cell system. Wu *et al.* [21] also reported that 247 genes were 1.5-fold or more differentially expressed after fluoride treatment in rat incisor pulp tissue. Up-regulation of *period circadian clock 1* (*Per1*) and down-regulations of *calpastatin* (*Cast*) and *programmed cell death 4* (*Pdcd4*) could be detected in ROE2 cells treated with NaF. Moreover, Sun *et al.* [22] identified 97 genes whose expressions were changed >2.0-fold in the sperm of mice treated with fluoride. Only 2 genes, *A kinase* (PRKA) *anchor protein 12* (*Akap12*) and *calcium/calmodulin-dependent protein kinase 1*, *alpha* (*Camkk1*), were found to be down-regulated in our NaF-treated cells. Therefore, almost all of the genes that were differentially regulated and identified here were not observed in these three microarray examinations [20–22].

## 4. Experimental Section

### 4.1. Cell Culture

Recently, we succeeded in establishing the immortalized oral epithelial cell line ROE2 from fetal transgenic rats harboring the temperature-sensitive simian virus 40 large T-antigen [25]. The cells with stratified epithelial-like morphology proliferated continuously at a permissive temperature of 33 °C and at an intermediate temperature of 37 °C. ROE2 cells were maintained in Dulbecco’s modified Eagle medium/Ham F-12 (1:1) (DMEM/F12) medium (Life Technologies Co., Grand Island, NY, USA) supplemented with 2% fetal bovine serum (FBS; Equitech-Bio Inc., Kerrville, TX, USA), 20 μg/L insulin, 20 μg/L transferrin, 1.22 μg/L ethanolamine, 91.4 ng/L sodium selenite and 10 μg/L epidermal growth factor (Sigma-Aldrich Co., St. Louis, MO, USA) in a collagen type I-precoated culture vessel (Asahi Glass Co., Ltd., Tokyo, Japan) at 33 °C [25]. The human malignant oral epithelial cell line HSC-3 was obtained from the Human Science Research Resources Bank of the Japan Health Sciences Foundation (Tokyo, Japan). HSC-3 cells were cultured in Eagle’s minimal essential medium (E-MEM; Wako Pure Chemical Industries Ltd., Osaka, Japan) supplemented with 10% FBS at 37 °C.

### 4.2. Incubation of Cells with the Compound

NaF (purity: 99.0%, Wako Pure Chemical Industries Ltd., Osaka, Japan) was dissolved in sterilized water. ROE2 cells were cultivated in DMEM/F12 medium supplemented with 2% FBS for 24 h at 37 °C, followed by incubation in DMEM/F12 medium supplemented with 2% FBS and the compound at final concentrations of 0 to 4 mM for 0–24 h at 37 °C. HSC-3 cells were cultured in E-MEM supplemented with 10% FBS and 2 mM NaF for 24 h at 37 °C. Cells treated with vehicle (sterilized water; 1% *v*/*v*) alone were used as a control.

### 4.3. Measurements of Cell Number, Cell Viability and Cell Death

For the trypan blue dye exclusion test, the number of cells excluding the dye was counted by using a hematocytometer. We also used Cell Count Reagent SF (Nacalai Tesque Inc., Kyoto, Japan), a water-soluble tetrazolium salt WST-8 [2-(2-methoxy-4-nitrophenyl)-3-(4-nitrophenyl)-5-(2,4-disulfophenyl)-2*H*-tetrazolium, monosodium salt] based assay, for this purpose. In short, cells were incubated in 110 μL of Daigo’s T medium (phenol red free, Wako Pure Chemical Industries Ltd.) containing 9.1% (*v*/*v*) of Cell Count Reagent SF in a 96-well cell culture plate at 37 °C. Two hours later, the produced formazan dye concentration was determined from the absorbance at 450 nm [54]. For measuring chromatin condensation, a Nuclear-ID™ Green Chromatin Condensation Kit (Enzo Life Sciences Inc., Farmingdale, NY, USA) was used. Finally, the stained samples were run on an Epics XL flow cytometer (Beckman Coulter Inc., Brea, CA, USA) [55].

### 4.4. Measurements of Protein Content and MMP

Protein content in cells was estimated by using a Pierce^®^ bicinchoninic acid (BCA) Protein Assay Kit (Pierce Biotechnology, Rockford, IL, USA). Bovine serum albumin was used as a standard. The optical density of the developed blue color was read at 570 nm. MMP was evaluated by JC-1 (5,5′,6,6′-tetrachloro-1,1′,3,3′-tetraethylbenzimidazolylcarbocyanine iodide) (JC-1 Mitochondrial Membrane Potential Assay Kit, Cayman Chemical Co., Ann Arbor, MI, USA). The fluorescence of monomeric (green) or aggregate JC-1 (red) was examined at an emission wavelength of 525 or 597 nm, respectively, under a fluorescence microscope.

### 4.5. Sodium Dodecyl Sulfate-Polyacrylamide Gel Electrophoresis (SDS-PAGE) and Western Blotting

After washing once with phosphate-buffered saline, the cells were scraped using a rubber policeman. Cells were dissolved in 50 mM Tris–HCl buffer (pH 7.4) containing 150 mM NaCl, 1% NP-40 and protease inhibitor cocktail (Nacalai Tesque, Inc.) and homogenized by an ultrasonic disruptor. Sodium dodecyl sulfate-polyacrylamide gel electrophoresis (SDS-PAGE) and Western blotting were carried out as described elsewhere [56,57]. The polyvinylidene difluoride membranes were incubated with the primary antibody at 4 °C overnight, and exposed to the peroxidase-conjugated secondary antibody at room temperature for 1 h. Immunoreactive proteins were visualized by a luminescent image analyzer using a chemiluminescence detection system. Primary antibodies used were as follows: anti-caspase-3 antibody (#9662, Cell Signaling Technology Inc., Beverly, MA, USA), anti-cleaved caspase-3 antibody (#9664, Cell Signaling Technology Inc.), anti-Hspa5 antibody (#3183, Cell Signaling Technology Inc.); anti-Ddit3 antibody (#2895, Cell Signaling Technology Inc.) and anti-glyceraldehyde 3-phosphate dehydrogenase (Gapdh) antibody (MAB374, Millipore Co., Temecula, CA, USA).

### 4.6. RNA Isolation

Total RNA was extracted from cells using an RNeasy Total RNA Extraction kit (Qiagen, Valencia, CA, USA) along with on-column DNase I treatment. RNA quality was analyzed using a Bioanalyzer 2100 (Agilent Technologies Inc., Santa Clara, CA, USA). RNA samples that had RNA integrity number values above 9.8 were considered acceptable.

### 4.7. Microarray and Pathway Analyses

A time course microarray analysis was carried out using a GeneChip^®^ system. RNA samples were obtained at 0, 3, 6 and 12 h post-vehicle treatment (0 mM NaF), and were obtained at 3, 6 and 12 h post-NaF (2 mM) treatment. For each time point, RNA from 3 independent experiments was pooled to generate 7 RNA pools. Seven Rat Genome 230 2.0 arrays, which were spotted with 31,099 probe sets (Affymetrix Inc., Santa Clara, CA, USA), were used. According to the manufacturer’s instructions, 500 ng of total RNA was used to synthesize cRNA with a GeneChip^®^ 3′ IVT Express Kit (Affymetrix, Inc.). Biotin-labeled cRNA was hybridized to the arrays at 45 °C for 16 h, and the arrays were stained with streptavidin phycoerythrin and scanned using a probe array scanner. The obtained hybridization intensity data were analyzed using GeneSpring^®^ GX (Agilent Technologies Inc.) to extract the significant genes. To examine gene ontology, including biological processes, cellular components, molecular functions, and gene networks, the obtained data were analyzed using Ingenuity^®^ Pathway Analysis tools (Ingenuity Systems Inc., Mountain View, CA, USA), a web-delivered application that enables the identification, visualization, and exploration of molecular interaction networks in gene expression data [23,24].

### 4.8. Real-Time Quantitative Polymerase Chain Reaction (qPCR)

Real-time qPCR was carried out on an Mx3000P real-time PCR system (Agilent Technologies Inc.) using SYBR PreMix ExTaq (Takara Bio Inc., Shiga, Japan) or Premix Ex Taq (for the use of TaqMan probes; Takara Bio Inc.) according to the manufacturer’s protocols. The reverse transcriptase reaction was carried out with total RNA by using a random 6 mers and an oligo dT primer. Based on the database, the specific PCR primers and probes were designed (Table S5). Each mRNA expression level was normalized with respect to the mRNA expression of *Gapdh* [23,24].

### 4.9. siRNA Transfection

ROE2 or HSC-3 cells were incubated in Opti-MEM^®^ I Reduced Serum Medium (Life Technologies Co.) containing Lipofectamine™ RNAiMAX (Life Technologies Co.) and 40 or 20 nM siRNA for 6 h at 37 °C, respectively, and were cultured in each standard culture medium for 42 h at 37 °C. The siRNAs were synthesized by Nippon Gene Co., Ltd. (Toyama, Japan) (Table S6). Luciferase siRNA was used as a negative control siRNA [55].

### 4.10. Statistical Analysis

Data are shown as the means ± standard deviations for at least 3 independent experiments except in the case of the microarray experiment. Statistical analysis was analyzed using Student’s *t*-test and *p* values less than 0.05 were regarded as statistically significant.

## 5. Conclusions

In conclusion, knowledge of the changes in gene expression and gene networks will provide further insight into the molecular mechanisms of NaF-induced cell death accompanying ER stress in oral epithelial cells.

## Figures and Tables

**Table 1.** The function of cell death-related genes in Up-I and Up-II clusters.

Clusters Functions	Genes
**Up-I**	
Pro-cell death 1	*Atf3*, *Ddit3*, *Egr1*, *Fos*, *Fosb*, *Fosl1*, *Gch1*, *Hbegf*, *Icam1*, *Mmp10*, *Nedd9*, *Nr4a1*, *Ppp1r15a*, *Rassf1*, *Tnf*, *Trib3*, *Zfp36*, *VGF*
Anti-cell death 2	*Abcb4*, *Areg*, *Bdkrb2*, *Clcf1*, *Dusp1*, *Egr2*, *Gdf15*, *Herpud1*, *Il6*, *Itsn1*, *Nr4a2*, *Nr4a3*, *Plaur*, *Procr*, *Sema6a*, *Tslp*, *Vegfa*, *Zmynd15*

**Up-II**	
Pro-cell death	*Abcb1*, *Atf2*, *Casp4*, *Casp12*, *Cxcl12*, *Fem1b*, *Il23a*, *Mllt11*, *Nrg1*, *Per1*, *Slscr1*, *Ppif*, *S100a8*, *Smox*, *Surf1*, *Tfrc*, *Zc3h8*
Anti-cell death	*Alkbh1*, *Atf4*, *Atp2b1*, *Bcl2a1*, *Cd55*, *Cdhr1*, *Cflar*, *Csf3*, *Dnajb9*, *Ehd4*, *Hspa5*, *Hyou1*, *Itgav*, *Lin7c*, *Lonp1*, *Mafk*, *Manf*, *Naa30*, *Nabp1*, *Odc1*, *Plaur*, *Rbbp6*, *Rbpj*, *Slc1a1*, *Slc25a19*, *Slc29a2*, *Sqstm1*, *Thbd*

1 Genes that are reported to induce cell death;

2 Genes that are reported to prevent cell death.
